# Radiation damage in room-temperature data acquisition with the PILATUS 6M pixel detector

**DOI:** 10.1107/S090904951100968X

**Published:** 2011-04-09

**Authors:** Chitra Rajendran, Florian S. N. Dworkowski, Meitian Wang, Clemens Schulze-Briese

**Affiliations:** a Swiss Light Source at Paul Scherrer Institute, CH-5232 Villigen, Switzerland

**Keywords:** room-temperature data collection, PILATUS 6M, dose rate, radiation damage

## Abstract

The first study of room-temperature macromolecular crystallography data acquisition with a silicon pixel detector is presented, where the data are collected in continuous sample rotation mode, with millisecond read-out time and no read-out noise. Several successive datasets were collected sequentially from single test crystals of thaumatin and insulin. The dose rate ranged between ∼1320 Gy s^−1^ and ∼8420 Gy s^−1^ with corresponding frame rates between 1.565 Hz and 12.5 Hz. The data were analysed for global radiation damage. A previously unreported negative dose-rate effect is observed in the indicators of global radiation damage, which showed an approximately 75% decrease in *D*_1/2_ at sixfold higher dose rate. The integrated intensity decreases in an exponential manner. Sample heating that could give rise to the enhanced radiation sensitivity at higher dose rate is investigated by collecting data between crystal temperatures of 298 K and 353 K. UV-Vis spectroscopy is used to demonstrate that disulfide radicals and trapped electrons do not accumulate at high dose rates in continuous data collection.

## Abstract 2

Observations of the dose-rate effect in continuous X-ray diffraction data acquisition at room temperature are presented.

## 1. Introduction

Radiation damage to biological crystals during synchrotron data collection is a major obstacle in macromolecular structure determination. While cryogenic cooling significantly reduces the detrimental effects of ionizing radiation, damage is commonly observed in the X-ray data collected at the third-generation synchrotron sources, even at cryogenic temperatures. The situation is much less favourable at room temperature (RT). The dose limit (20 MGy) at which the diffraction intensity is predicted to fall to at least half of its initial value at cryo-temperatures was derived by Henderson (1990 ▶) for cryo-EM and experimentally confirmed for X-ray protein crystallography by Owen *et al.* (2006 ▶) to be 43 MGy, and has also been investigated by other researchers (Teng & Moffat, 2000 ▶, 2002 ▶; Kmetko *et al.*, 2006 ▶). In the case of RT data collection the reported dose limits are almost two orders of magnitude lower (Blake & Phillips, 1962 ▶; Nave & Garman, 2005 ▶; Southworth-Davies *et al.*, 2007 ▶; Warkentin & Thorne, 2010 ▶).

Despite significant progress in rational methods for cryoprotection in macromolecular crystallography (Alcorn & Juers, 2010 ▶), there are frequently cases in which crystals become disordered or introduce internal lattice changes during the cooling process. Furthermore, some virus crystals are not easily cooled and exhibit high mosaicity (Barker *et al.*, 2009 ▶). In such circumstances, RT data collection can play a major role and several recent studies have investigated means to reduce radiation damage at RT. The use of radical and electron scavengers was reported to have a beneficial effect (Barker *et al.*, 2009 ▶). Moreover, a measurable positive dose-rate effect was reported at very low dose rates (Southworth-Davies *et al.*, 2007 ▶) as well as at moderate dose rates (Barker *et al.*, 2009 ▶).

Recently, the high frame rate of 12.5 Hz in combination with continuous data acquisition mode and a dynamic range of 20 bits, supported by the PILATUS pixel detector, has offered the opportunity for data to be collected with unprecedented speed. In continuous data acquisition the total acquisition time is determined by the dose rate in an inverse manner, and an increased dose rate shortens the data collection time. Depending on the point-group symmetry a complete data set can be acquired in only a few seconds. This paves the way for a new form of kinetic crystallography (Bourgeois & Royant, 2005 ▶; Colletier *et al.*, 2008 ▶), where snapshots of the reaction mechanism along the enzymatic pathway can be captured, without the need to cryo-trap intermediates, provided that the reaction kinetics in the crystal is of appropriate rate. In a prototype experiment the reversible transfer reactions of coenzyme A carriers from CoA-thioesters to free acids by CoA transferase (Berthold *et al.*, 2008 ▶) were investigated (Schneider, 2008 ▶). The highest achievable temporal resolution in this kind of experiment obviously depends on the maximal tolerable dose rate, but little is currently known about the dose-rate dependence of radiation damage at RT for dose rates typically available at third-generation synchrotrons.

Greater knowledge of the RT dose-rate dependence would also be beneficial for other purposes. In virus crystallography it is a common strategy to work at maximum dose rate (Stuart, 2011 ▶; Rey, 2011 ▶), as a way of reducing secondary radiation damage, although optimal dose rates are not reported in the literature. RT data collection is also ideally suited to establish the crystal diffraction properties in the absence of a possible degradation owing to the cryo-protocol (Kurinov & Harrison, 1995 ▶; Garman, 1999 ▶; Kriminski *et al.*, 2002 ▶; McFerrin & Snell, 2002 ▶; Juers & Matthews, 2004 ▶; Lovelace *et al.*, 2006 ▶). This is particularly true for *in situ* diffraction screening (Bingel-Erlenmeyer *et al.*, 2011 ▶), which reduces crystal manipulation to an absolute minimum, and which is becoming available at more and more synchrotron radiation facilities. In cases where suitable cryo-protocols cannot be devised, or in order to scrutinize cooling-induced structural changes, the collection of complete data sets at RT is possible when optimized data acquisition strategies are applied. The properties of counting hybrid pixel detectors offer several advantages to this end. The very low mosaic spread can be exploited by data collection in fine ϕ-sliced mode at relatively low dose per frame and the resulting small spot size on the detector, particularly at low-divergence undulator beamlines, is ideally matched by the very narrow point-spread function (Hülsen *et al.*, 2006 ▶).

On the other hand many crystallographers avoid RT data collection these days because of the tedious work of mounting the crystal with glass capillaries and their incompatibility with beamline automation. However, with the advent of the Mitegen RT system (http://www.mitegen.com/), mounting of crystals for RT data collection is as easy as the normal loop mounting for data collection at cryogenic temperatures (Kalinin *et al.*, 2005 ▶). Alternatively, a device for humidity control of macromolecular crystals mounted in cryoloops can be used for RT data collection (Sanchez-Weatherby *et al.*, 2009 ▶).

The main aim of the present study was to investigate the dose-rate dependence of radiation damage at RT and to determine dose limits for RT crystallography in continuous data acquisition mode, with the objective of minimizing global damage effects and to provide an estimate of the temporal resolution achievable in kinetic crystallography.

## 2. Materials and method

### 2.1. Crystallization

Insulin and thaumatin were obtained from Sigma Aldrich and crystallized *via* the hanging-drop vapour diffusion method. The reservoir solution contained 0.01 *M* Na_3_EDTA and 0.2 *M* Na_3_PO_4_/Na_2_HPO_4_ for insulin (Dodson *et al.*, 1978 ▶). Thaumatin was crystallized from 2 *M* NaK-tartarate (Ko *et al.*, 1994 ▶). The protein concentration was 20 mg ml^−1^ for both samples. The drop contained 2 µl of well solution and 2 µl of protein solution. The crystals were obtained in one to two days at RT. The space group of insulin was *I*2_1_3 and of thaumatin was *P*4_1_2_1_2. The sizes of the insulin crystals were approximately 0.15 × 0.15 × 0.05 mm and those of thaumatin crystals were approximately 0.3 × 0.2 × 0.2 mm. The solvent contents for insulin and thaumatin were 63% and 57%, respectively.

### 2.2. Crystal mounting

The Mitegen RT system (Kalinin *et al.*, 2005 ▶) was used for the crystal mounting. It has a flexible transparent thin-wall PET tube pre-sealed at one end. The PET tube was cut to the desired length, then filled at the closed end with a small volume of mother liquor from the well to minimize dehydration and placed over the crystal onto the base, without disturbing the crystal. Either regular nylon (http://www.hamptonresearch.com/) or litho-loops (http://www.mitegen.com/) were used for crystal mounting. Owing to their morphology, the thaumatin crystals were preferentially aligned with their long axis parallel to the spindle, such that the exposed crystal volume remains constant during sample rotation. For cryogenic measurement the crystals were mounted with the regular nylon loop and a cryoprotectant of 15% glycerol was added before freezing the crystals in liquid nitrogen.

### 2.3. Dose calculations

The flux was determined by using a silicon pin diode as described by Owen *et al.* (2009*a* ▶). The beam size was determined by knife-edge scans. The beam size was 0.1 mm × 0.09 mm (v × h) and was of Gaussian shape in the horizontal and of flat-top shape in the vertical direction. Dose calculations were performed using the program *RADDOSE* (Murray *et al.*, 2004 ▶; Paithankar *et al.*, 2009 ▶), which takes the beam parameters and crystal composition into account to calculate the absorbed dose. It should be noted that the effective dose to the crystal is in general smaller than the values calculated by *RADDOSE* since the beam was smaller than the crystals (Schulze-Briese *et al.*, 2005 ▶), particularly in the case of thaumatin. However, since the crystals were of similar size (within the four dose rates tested for each test system), a relative comparison of the radiation sensitivity is possible. Only the thaumatin crystal measured at 6.25 Hz had a smaller cross section (∼150 µm × 150 µm) than all the other thaumatin crystals. Consequently, less fresh material is moved into the beam when this crystal is rotated than in the case of the larger crystals. In order to account for this difference, the dose of the 6.25 Hz data collection calculated from *RADDOSE* has been corrected (increased) by a factor of 1.35. The doses quoted in the following sections have been adjusted accordingly. On the other hand, insulin and thaumatin crystals not only differ in size but also in morphology and no attempt was undertaken to compare the results on an absolute scale.

### 2.4. Experimental design and data collection

The experiment was performed at four different dose rates, where the dose per frame was kept similar and only the exposure time per frame and the dose rates were varied, while all the other parameters were kept constant. The dose rates investigated were between ∼1320 Gy s^−1^ and ∼8420 Gy s^−1^ (Table 1 ▶). Note that the flux used in the experiments is one to three orders of magnitude below flux levels achievable at modern undulator beamlines.

In each successive experiment the exposure time was doubled and in turn the dose rate was reduced twofold. This kept the dose received by each frame constant, while the data acquisition frequency was reduced by half. The experiments are labeled as I_12 or T_12, where I stands for insulin, T for thaumatin and 12 indicates the detector was operated at 12.5 Hz frame rate. Therefore I_12 means an insulin crystal measured at 12.5 Hz frequency, and T_12 means a thaumatin crystal measured at 12.5 Hz frequency. Similarly 6, 3 and 1 indicate the frequencies 6.25 Hz, 3.125 Hz and 1.5625 Hz, respectively. The read-out time of the PILATUS 6M, during which the detector is not sensitive to X-rays, amounts to 2.3 ms per frame at all data acquisition frequencies. The oscillation angle was 0.5° for all the experiments. Successive datasets were collected from a single crystal, *i.e.* the sample was rotated continuously. For insulin, a total of 720 frames and for thaumatin 1440 frames were collected. The sample temperature during data collections was 298 K, if not indicated otherwise.

For the insulin and thaumatin crystals referred to in Tables 2 ▶ and 3 ▶, data collection was performed at 100 K. The flux during the measurement was 4.8 × 10^10^ photons s^−1^. The calculated dose was 1.4 MGy and 2.9 MGy for insulin and thaumatin, respectively. The number of frames per dataset was 96 for insulin and 192 for thaumatin, with an oscillation angle of 0.5°.

The elevated temperature data collection for insulin crystals was carried out at temperatures of 303, 313, 323, 333 and 343 K. The flux during the experiment was ∼2.5 × 10^10^ to 2.9 × 10^10^ photons s^−1^. The number of frames per dataset was 90 with 0.5° oscillation angle. The experiments were labeled as I_303, where I stands for insulin and 303 indicates that the dataset was collected at a temperature of 303 K. Similarly, I_313, I_323, I_333 and I_343 indicate that the datasets were collected at 313, 323, 333 and 343 K for insulin crystals, respectively. The successive dataset collected from the same insulin crystal I_343 was called I_343-2.

### 2.5. Data processing

Data processing of all RT data sets was carried out using the script *go.com* (SLS internal development; Wang, 2011 ▶). The *go.com* script combines the fast indexing of *LABELIT* (Sauter *et al.*, 2004 ▶), the robust data processing of *XDS* (Kabsch, 2010 ▶) and various data quality assessment programs such as *POINTLESS* and *SCALA* (Evans, 2006 ▶), and *PHENIX.XTRIAGE* (Adams *et al.*, 2010 ▶) to process X-ray diffraction images in an automatic manner. In addition, the script fully exploits the fast computing framework of the SLS protein crystallography beamlines to run *XDS* in parallel mode (using up to 32 processors). It takes less than one minute to process 90° of data, determine space groups, convert reduced data to mtz format, and present a summary of the data processing statistics. This fast and reliable data processing allows diffraction data quality and radiation damage to be analyzed objectively during the experiment. The immediate assessment of the data quality, in particular of the completeness, is of utmost importance in RT data acquisition, because of the significant higher radiation sensitivity as compared with cryogenic temperatures, which also limits the possibility of taking test shots to characterize the diffraction quality prior to the data acquisition. During processing, for insulin data, wedges of 90 images (45°) were considered as one dataset, and in the case of thaumatin, wedges of 120 images (60°) were considered as one dataset, resulting in 8 and 12 subsequent data sets for insulin and thaumatin, respectively. The parameters indicated in Tables 2–6 were extracted from the *XDS* processing output files CORRECT.LP. The data statistics shown in Tables 2 ▶ and 3 ▶ are from experiments carried out at cryogenic temperatures (100 K). The cryogenic data were collected at 0.5° oscillation angle, using 96 images for processing insulin and 120 images for thaumatin. The statistics for one dataset of each collected from insulin and thaumatin crystals at cryogenic temperature (100 K) are presented in Tables 2 ▶ and 3 ▶ to allow easy comparison of the data statistics with those of data taken at RT. The data statistics from the datasets for insulin at elevated temperatures (303, 313, 323, 333 and 343 K) are shown in Table 4 ▶.

### 2.6. UV-Vis absorption spectroscopy experiments

UV-Vis absorption spectroscopy experiments can reveal the presence of radiolytic products and radicals. Since their accumulation at RT may be dose-rate dependent, the optical density (OD) of an insulin crystal was measured in a separate experiment by means of the co-axial microspectrophotometer installed at SLS beamline X10SA (Owen *et al.*, 2009*b* ▶). The size of the X-ray beam was ∼110 µm × 90 µm and the dose rate was calculated to be 9.0 × 10^3^ Gy s^−1^ (*RADDOSE*; Murray *et al.*, 2004 ▶). The temporal evolution of the signal at 400 nm and 600 nm was measured at 298 K, and also at 100 K for reference. The absorbance was analyzed at 400 nm and at 520–620 nm, corresponding to disulfide radical anions and trapped solvated electrons, respectively (McGeehan *et al.*, 2009 ▶).

### 2.7. Sample heating experiment

Since the temperature of the sample was not regulated and not monitored during the experiments, elevated dose rates may have given rise to sample heating, which would eventually compromise the diffraction quality of the sample, enhancing the damage induced by the radiation. Although the temperature rise calculated with *RADDOSE* is less than 0.1 K at all dose rates, an experiment was carried out in order to determine the maximal temperature at which insulin crystals diffract. Data were collected at elevated temperatures ranging from 303 to 353 K. A conventional hot gun was used to blow warm air at the sample mounted using the Mitegen RT system. The temperature was measured with a thermocouple, which was kept within ∼3 mm distance from the sample. The state of the insulin crystals before and after data collection at the different temperatures was observed with the sample alignment microscope (images are presented in the supplementary material, Fig. S1^1^). Each dataset corresponded to 45° (90 images of 0.5° oscillation angle), with 0.64 s per frame exposure time at a flux of ∼2.5 × 10^10^ to 2.9 × 10^10^ photons s^−1^, corresponding to the settings of the 1.5625 Hz data collection. The beam size was 100 µm × 80 µm, resulting in a dose of 0.09 MGy per data set (∼1540 Gy s^−1^). The data statistics for all the datasets at elevated temperatures are shown in Table 4 ▶. A successive dataset was collected at 343 K (I_343-2) to calculate *D*_1/2_.

## 3. Results

The data collection statistics of insulin and thaumatin with different dose rates at RT are shown in Tables 5 ▶ and 6 ▶, respectively. To analyse the effect of radiation damage, several indicators of global radiation damage, such as the scaled intensities *I* of all the reflections in a dataset, the redundancy independent *R*-factor *R*_meas_, the mean of the reflection intensities *I* divided by its standard deviation *I*/σ(*I*), the Wilson *B*-factor and the crystal mosaicity were monitored as a function of absorbed dose *D*. Data collection statistics of insulin and thaumatin crystals at cryogenic temperature are reproduced in Tables 2 ▶ and 3 ▶, respectively (Müller, 2010 ▶). Comparison of the statistics for the first RT and the dataset of cryogenic (100 K) datasets show that the mosaicity is comparatively lower at RT. It is also clear that usable datasets of reasonable quality could be collected at RT.

### 3.1. Relative intensity *versus* absorbed dose at different dose rates

**Figure 1:**
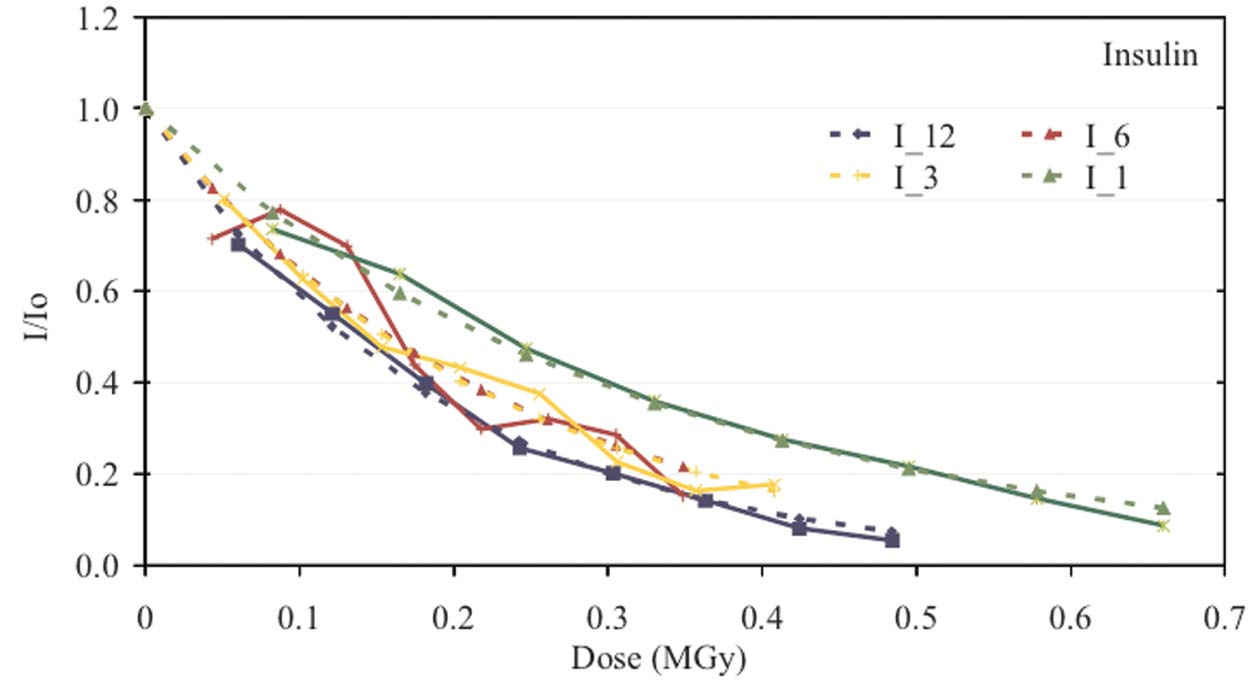
Plot of the relative summed intensity decay (〈*I*/*I*_0_〉) as a function of absorbed dose for insulin crystals at dose rates I_12 (∼8420 Gy s^−1^, blue), I_6 (∼3030 Gy s^−1^, red), I_3 (∼1775 Gy s^−1^, yellow) and I_1 (∼1430 Gy s^−1^, green). The resolution range is between 4.8 and 1.6 Å.

An exponential decay of intensity with dose was observed for both insulin and thaumatin crystals. This first-order character of the decay process was also observed with several previous studies carried out at significantly lower dose rates (Southworth-Davies *et al.*, 2007 ▶, and references therein). The relative summed intensity of the successive data sets was extrapolated to 0-dose using the exponential model *I*(*D*) = *I*_0_exp(−κ*D*). The normalized relative intensity *I*/*I*_0_ is plotted against absorbed dose in Figs. 1 ▶ and 2 ▶, with *I* referring to the mean intensity of a dataset and *I*_0_ to the extrapolated intensity at 0-dose for insulin and thaumatin crystals, respectively. The exponential fit to the data is superimposed on the data points. The comparison of the intensity decay at the different dose rates studied shows that the intensity decay rate is increasing with higher dose rates. This trend is observed to be similar in both test systems.

**Figure 3:**
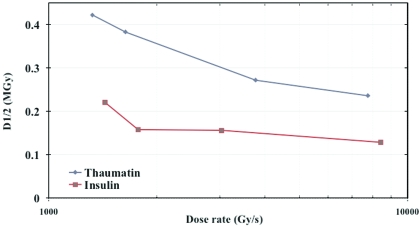
*D*_1/2_ as a function of dose rate for insulin and thaumatin crystals.

*D*_1/2_ is the dose at which the intensity of the diffraction pattern is reduced to half its original value and was derived from the exponential fits to the measured data. For thaumatin at dose rates of ∼1320, ∼1635, ∼3770 and ∼7760 Gy s^−1^, the *D*_1/2_ values are 0.42, 0.38, 0.27 and 0.24 MGy, respectively. Similarly, the *D*_1/2_ values for insulin at dose rates of 1430, 1775, 3030 and ∼8420 Gy s^−1^ are 0.22, 0.16, 0.15 and 0.13 MGy, respectively. Fig. 3 ▶ shows *D*_1/2_ as a function of dose rate.

### 3.2. Relative Wilson *B*-factor

**Figure 4:**
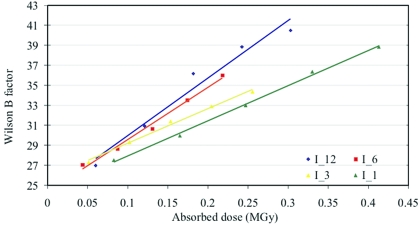
Plot of the change in Wilson *B*-factor of successive datasets for insulin crystals I_12, I_6, I_3 and I_1.

The plots of Wilson *B*-factors at different dose rates for both the insulin and thaumatin crystals (Figs. 4 ▶ and 5 ▶) against absorbed dose show that the *B*-factor increases linearly with dose, consistent with the observed decrease of the mean intensities. This result is in contrast to previous studies, which suggested that the *B*-factor increase was not consistently linear at RT (Southworth-Davies *et al.*, 2007 ▶). Furthermore, the observed *B*-factor increase is larger at high dose rates than at low dose rates. Linear fit analysis of the *B*-factor *versus* dose rates is shown in Table 7 ▶. There is an excellent correlation between the relative *B*-factor Δ*B*/Δ*D* (Å^2^ MGy^−1^) and the exponential decay constant κ as a function of dose rate, indicating that the *B*-factor is a good metric to monitor radiation damage in RT experiments at high flux rates. The deviation from linearity at high doses may have its origin in the high level of radiation damage, which caused a significant reduction of the resolution limit of the observed diffraction.

### 3.3. Redundancy independent *R*-factor

In accordance with the other indicators, the rise of *R*_meas_ at higher dose rate is more pronounced than at lower dose rate. In insulin and thaumatin crystals, *R*_meas_ increases at all measured dose rates in a clearly exponential manner (χ^2^ values are typically three times higher for a linear than for an exponential fit at all data acquisition rates apart from 3.125 Hz, where the difference is smaller). Similar to the behaviour of the *B*-factor, some curves start to fluctuate above a certain dose. In thaumatin crystals the very steep increase in *R*_meas_ of the data set T_6 could be due to the smaller dimension of this crystal. Figs. S2 and S3 (supplementary material) summarize *R*_meas_ *versus* absorbed dose at different dose rates for insulin and thaumatin, respectively.

### 3.4. Mean intensity over noise

The 〈*I*/σ(*I*)〉 values decrease with increasing dose at all studied dose rates, in agreement with all other indicators of global radiation damage. Values of 〈*I*/σ(*I*)〉 for data measured at higher angular speed are lower than for data recorded at lower angular speed. An increase in exposure time by a factor of four increases 〈*I*/σ(*I*)〉 by approximately a factor of 1.5, for the same dose to the crystal. In insulin crystals the increase in 〈*I*/σ(*I*)〉 between high and low angular speed is ∼1.5-fold (Fig. S4 of supplementary material) and in the case of thaumatin crystals the rise is ∼1.4-fold (Fig. S5 of supplementary material).

### 3.5. Mosaicity

The mosaicities of the first data set of each series are remarkably narrow and range from 0.02° to 0.04°, well below typical values obtained for optimally cryo-cooled insulin and thaumatin crystals (0.05° to 0.2°, *cf.* Tables 2 ▶ and 3 ▶; Müller, 2010 ▶)^2^. The increase in mosaicity with dose varies but is in general higher for high dose rates for both insulin and thaumatin (Figs. S6 and S7, respectively, in supplementary material). The mosaicity grows exponentially with dose, with a relatively small increase up to approximately 0.3 × 10^6^ Gy followed by a more rapid crystal deterioration at higher doses. The rise in mosaicity for the insulin data set I_12 is from 0.027° to 0.28°, *i.e.* a tenfold increase between the first and the last dataset. In the case of thaumatin, the rise in mosaicity is from 0.04° to ∼0.2°, which is fivefold for the high dose rate (∼7760 Gy s^−1^).

### 3.6. Visible change

**Figure 6:**
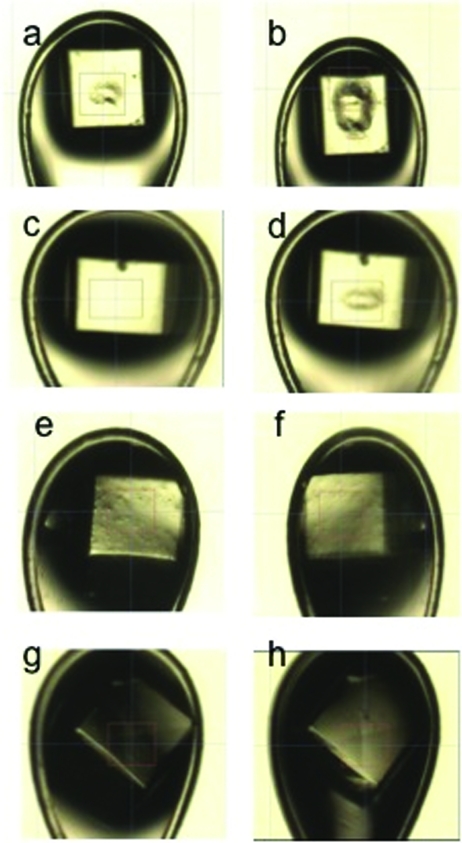
A set of example images showing the state of insulin crystals before and after RT data collection (360°): (*a*) I_12 after data collection, (*b*) I_12, 15 min after data collection, (*c*) I_6 before data collection, (*d*) I_6 after data collection, (*e*) I_3 before data collection, (*f*) I_3 after data collection, (*g*) I_1 before data collection, (*h*) I_1 after data collection.

The visible changes in the samples were observed with the sample alignment microscope and are presented in Fig. 6 ▶ for insulin crystals. The visible damage to the sample was greater at higher dose rate than at the lower dose rate. At dose rates in excess of 3000 Gy s^−1^ a hole-like structure is visible in the beam area after the data acquisition (*c.f.* Figs. 6*a*, 6*b* and 6*d* ▶). Leaving the crystal unexposed on the goniometer for 15 min after irradiation with 8420 Gy s^−1^ results in a significant further growth of the damaged crystal volume (Fig. 6*b* ▶). No visible changes are observed at dose rates below 3000 Gy s^−1^ (Figs. 6*f* and 6*h* ▶). Visually, thaumatin crystals displayed the same dose-rate-dependent behaviour.

### 3.7. UV-Vis absorption spectroscopy

UV-Vis absorption spectroscopy experiments were carried out in order to determine the potential accumulation of solvated electrons (520–620 nm) and disulphide radical anions (400 nm) (McGeehan *et al.*, 2009 ▶). While the control experiment at cryogenic temperatures indicated a clear build up of disulphide radical anions at a dose rate of ∼9000 Gy s^−1^ and a very weak signal owing to trapped electrons, no indication of either of the radiolytic products could be detected at RT (Figs. S8*a*, S8*b* and S8*c* of supplementary material).

### 3.8. High-temperature experiment and unit-cell expansion

The high-temperature experiment revealed that crystallized insulin is remarkably stable. The diffraction quality only decreased slightly up to 313 K and then deteriorated quickly with further temperature increase. While a complete data set could still be indexed and processed at 343 K, no diffraction was observed at 353 K. The indexed and processed data statistics are shown in Table 4 ▶. From the statistics it can be seen that the resolution decreases as the temperature increases from 303 to 343 K. The *R*_meas_, Wilson *B*-factor and mosaicity increase and the corresponding 〈*I*/σ*I*〉 values decrease as the temperature is increased from 303 to 343 K. All the datasets were collected at the same dose rate (1540 Gy s^−1^). These observations clearly indicate that the quality of data decreases with increasing temperature. At 343 K the data start to get worse, which agrees well with the dissociation and unfolding temperature of insulin in solution, which is reported to be 343 K (Huus *et al.*, 2005 ▶). The calculated *D*_1/2_ at 343 K was ∼0.15 MGy, based on the statistics from the two successive datasets collected from the insulin crystal I_343 (Table 4 ▶).

The increase of the lattice constant as a function of temperature can be fitted with a linear model, revealing an expansion coefficient α of 7.9 × 10^−5^ K^−1^. In contrast to the previous study by Southworth-Davies *et al.* (2007 ▶), in our experiments a systematic dose rate and dose dependence of the unit-cell volume was observed for both of the proteins during the course of the data collection, although the unit cell was the same for all the crystals at the beginning of the data collection. At the lowest dose rate the volume shrinks by almost 1% during the course of the data acquisition (I_1 and T_1, Fig. S9 of supplementary material). At higher dose rates the decrease is less pronounced and for I_12 a unit-cell expansion is observed beyond a dose of 0.3 × 10^6^ Gy (Fig. S10 of supplementary material).

## 4. Discussion

This paper reports a previously unobserved negative dose-rate effect at intermediate dose rates in continuous RT data acquisition with a counting pixel detector. Insulin and thaumatin crystals were studied at dose rates ranging from 1320 to 8420 Gy s^−1^ and the diffraction data were analysed in terms of global radiation damage. All observed trends were identical for both samples. The principal findings are a decrease of the dose required to half the integrated diffraction intensity, *D*_1/2_, and an increase of the Wilson *B*-factor and of the rate of increase of *R*_meas_ with dose rate. UV-Vis spectroscopy did not reveal an accumulation of radiolytic products at the highest dose rates studied. Diffraction experiments at elevated temperatures showed insulin to diffract up to 343 K. The crystal mosaicity was found to be very low, but compared with vitrified crystals it grew in an exponential manner with dose, again at increasing rates at higher dose rates. The small increase of the mosaicity up to 0.3 × 10^6^ Gy could have its origin in the overestimation of the intrinsic mosaicity as a consequence of the relatively coarse oscillation angle of 0.5° as well as of the beam divergence (0.02° × 0.004°)^3^. The low crystal mosaicity is also the origin of the lower 〈*I*/σ(*I*)〉 value of data sets collected at higher acquisition frequency. At an angular speed of 6.5° s^−1^ the reciprocal lattice point crosses the Ewald sphere in a few milliseconds, being sensitive to beam intensity and position instabilities which are averaged out at lower angular speed. The increase in the mosaicity with higher absorbed doses thus partly compensates for the disorder-induced decrease of 〈*I*/σ(*I*)〉 as observed for insulin and thaumatin at 12.5 Hz acquisition frequency (*c.f.* Figs. S4 and S5 of supplementary material). Finally, the change in the unit-cell volume also depends on the dose rate, shrinking at lower and growing at higher dose rates.

Dose rate effects in protein crystallography have been studied previously, both at cryogenic temperature (Ravelli *et al.*, 2002 ▶; Sliz *et al.*, 2003 ▶; Owen *et al.*, 2006 ▶; Leiros *et al.*, 2006 ▶) and at RT (Southworth-Davies *et al.*, 2007 ▶; Barker *et al.*, 2009 ▶). Southworth-Davies *et al.* (2007 ▶), reporting on dose-rate effects between 6 and 10 Gy s^−1^ at RT, showed a positive linear relationship between *D*_1/2_ and the dose rate for lysozyme crystals, *i.e.* an inverse dose-rate effect. The group further reported a *D*_1/2_ of 0.9 MGy for lysozyme measured in a synchrotron experiment at a dose rate of 2800 Gy s^−1^ (Barker *et al.*, 2009 ▶), as compared with 0.38 to 1.63 MGy at 6 and 10 Gy s^−1^, respectively (Southworth-Davies *et al.*, 2007 ▶). A study of radiation damage to lipidic mesophases at RT by Cherezov *et al.* (2002 ▶) gave evidence for normal and inverse dose-rate effects for dehydrated and hydrated samples, respectively, resulting from different types of damage. The absolute *D*_1/2_ values presented in this study, ranging from 0.13 to 0.42 MGy, overlap those reported by Southworth-Davies *et al.* (2007 ▶) and Barker *et al.* (2009 ▶) (0.38–1.63 MGy), and with the results of Cherezov *et al.* (2002 ▶) (0.07–0.18 MGy). While the extrapolation to 0-dose reduces the *D*_1/2_ value as compared with a fit normalized to the first data set, other causes for deviations may have their origin in the details of the experiment, such as the precise beam profile, the ratio of crystal to beam size, *i.e.* unexposed crystal volume, possible dehydration, *etc.*, which cannot easily be tracked. Other major differences between this work and all other studies are the continuous data collection mode used for acquisition and the crystal types used for the study. In the continuous mode there is no time during which reactive species may recombine or decay, thereby limiting the maximal concentration. In summary, the results of this study in conjunction with the literature clearly indicate the existence of an optimal dose rate for data acquisition at RT between 10 Gy s^−1^ and 1400 Gy s^−1^.

Consideration of sample heating as a possible cause of enhanced damage at higher dose rates has been studied intensively at cryogenic temperature (100 K). The results indicate either no or only a small dose-rate effect (Ravelli *et al.*, 2002 ▶; Sliz *et al.*, 2003 ▶; Owen *et al.*, 2006 ▶; Leiros *et al.*, 2006 ▶). The dose-rate effect observed at cryogenic temperatures by Owen *et al.* (2006 ▶) is significantly smaller (10% decrease in *D*_1/2_ for tenfold flux increase) than in the present RT study, where a six-times-higher dose rate resulted in a reduction of *D*_1/2_ by 75%.

In summary, the absolute values of the half doses reported here (0.13 to 0.42 MGy) are in the upper range of the reported cryocooling protection factor, which has been calculated to be 70 (Nave & Garman, 2005 ▶) using a *D*_1/2_ (0.59 MGy) extracted from the RT work of Blake & Phillips (1962 ▶) on myoglobin and the 100 K limit of 43 MGy (Owen *et al.*, 2006 ▶). However, this protection factor is now known to vary between 26 and 113 times (Southworth-Davies *et al.*, 2007 ▶) for low-dose-rate data collections.

The nature of the decay of the integrated intensities is of first order (*c.f.* Figs. 1 ▶ and 2 ▶). In conjunction with the observed linear increase of the *B*-factor this could be indicative of the creation of increased disorder as opposed to a completely disorganized or amorphous fraction. The detailed analysis of the kinetics of the radiation-induced generation of a dis­ordered and an amorphous fraction, as presented by Hendrickson (1976 ▶) on the basis of the Blake & Phillips (1962 ▶) data, cannot be attempted here because the crystals are larger than the beam. Hence, varying fractions of unirradiated material contribute to the diffraction pattern when the crystal is rotated in the beam.

What is the origin of the negative dose-rate effect observed at intermediate dose rates as compared with the positive dose-rate effect at low dose rates? First, the experiments were performed with continuous sample rotation, whereas the experiments by Southworth-Davies *et al.* (2007 ▶) were carried out with 90 s detector read-out time during which the sample was not irradiated. It is possible that in the latter case the radiolytical products recombine during the read-out time and do not have a cumulative effect as observed in continuous data collection mode. On the other hand, the UV-Vis absorption spectroscopy experiment revealed no accumulation of hydrated electrons and disulphide radical anions at the highest dose rate studied here. It is, therefore, unlikely that accumulation of radiolytic agents causes the observed dose-rate effect in continuous data acquisition. One hypothesis for lower dose tolerance of the crystals at high dose rates is the more rapid temperature rise in the sample at high dose rates compared with low dose rates. Higher sample temperature increases the mobility of the radicals formed, thereby causing more secondary damage as they diffuse. Although the temperature rise calculated in *RADDOSE* (Murray *et al.*, 2004 ▶) simulations as well as from comparison with published experimental results (Snell *et al.*, 2007 ▶) was expected to be small, sample heating was investigated by collecting data at elevated temperatures. Insulin crystals diffracted up to 343 K and, even at this temperature, a complete dataset could be collected, indexed and processed. The dose rate used for the elevated temperature data collection was ∼1540 Gy s^−1^, which is comparable with the low dose rate (1.5625 Hz, ∼1430 Gy s^−1^) in our RT study. The approximate *D*_1/2_ at 343 K for insulin was 0.15 MGy, which is ∼1.5 times less than the *D*_1/2_ at RT, which is 0.22 MGy. The decay of the diffraction intensities at 343 K occurred at much lower dose than at RT, confirming a temperature-induced sensitization. The unit cell was refined to almost the same value for the first data sets collected at the different dose rates with very low standard deviation (78.9 ± 0.046 Å). At the start of the data collection the absolute values of the unit-cell parameters were the same and there is no dependence of the initial unit-cell size on the dose rate. The unit cell measured at highest dose rate is actually the smallest. Furthermore, the unit-cell volume decreases as the data acquisition series proceeds and then some of them increase again (*cf.* Figs. S9 and S10 of supplementary material).

This latter finding suggests another possible explanation. The generation of hydrogen was previously suggested by Meents *et al.* (2010 ▶) to be the probable origin of global radiation damage at cryogenic temperatures. Several observations support the idea that molecular hydrogen may contribute to the dose-rate effect at RT. The unit-cell volume shrinks at low dose rates, as it would be expected in the case of mass loss due to hydrogen diffusion out of the crystal. On the other hand, at high dose rates (>3000 Gy s^−1^) the formation of a hole in the region of the beam footprint is observed, which still continues to expand after the end of the data acquisition. At this dose rate the unit-cell volume increases slightly and the mosaicity shows a significantly faster increase than at lower dose rates.

## 5. Conclusions

The dose-rate effect in continuous data acquisition with a hybrid pixel detector was studied at RT at medium dose rates. The present results in combination with data in the literature suggest an optimal dose rate for RT data acquisition between 10 and 1400 Gy s^−1^. The variation in *D*_1/2_ with dose rate could be due to the different crystal species and/or different crystallization conditions and has important consequences for RT data acquisition experiments and strategies. First, it does not seem to be the optimal strategy to maximize the dose rate to maximize the amount of data that can be collected from a single crystal. Second, the temporal resolution achievable in kinetic experiments at RT may be severely curbed by the maximal tolerable dose rate. Another limitation of the maximal possible temporal resolution stems from the observed degradation of 〈*I*/σ(*I*)〉 with increasing angular speed. Taken together, the temporal resolution in kinetic crystallography at RT using current protocols and technology is therefore estimated to be of the order of 5 s. All global indicators of radiation damage with the exception of 〈*I*/σ(*I*)〉 show a very consistent behaviour throughout this study and may be used to quantify RT radiation damage.

Future work should address the question of the influence of lowering the temperature (*e.g.* to 277 K or even to 203 K) on the damage rates as well as a means to slow down reaction kinetics, while still preserving the possibility of conformational changes and of diffusion processes (Warkentin & Thorne, 2010 ▶). Investigation of the nature of the observed enhanced damage rates at high dose rates, and on different crystal types, deserves additional work. Delays between the acquisitions of data sets would be a strategy to investigate the effect of possible diffusion effects. In the future, global damage to the crystal structure should be studied in a wider range of dose rates.

On the other hand, the results clearly show that RT data acquisition at synchrotrons is possible and that it benefits from the properties of the PILATUS pixel detector. In order to obtain optimal statistics, the data should be collected with low dose rate. At cryogenic temperatures and at RT, data should be collected with fine ϕ-slicing, with Δϕ being equal to 1/4 to 1/2 of the mosaicity. However, at RT this suggests typical oscillation angles of 0.005 to 0.05°. The very low mosaicity gives rise to very small beam spots on the detector, which result in optimal signal-to-noise ratio owing to the very narrow point-spread function of hybrid pixel detectors. The next generation of pixel detectors currently under development at PSI, called EIGER, will support frame rates up to 10000 frames s^−1^ and hence will be ideally suited for both fine ϕ-sliced data acquisition and kinetic crystallography (Dinapoli, 2011 ▶).

## Supplementary Material

Supplementary material file. DOI: 10.1107/S090904951100968X/xh5025sup1.pdf

## Figures and Tables

**Figure 2:**
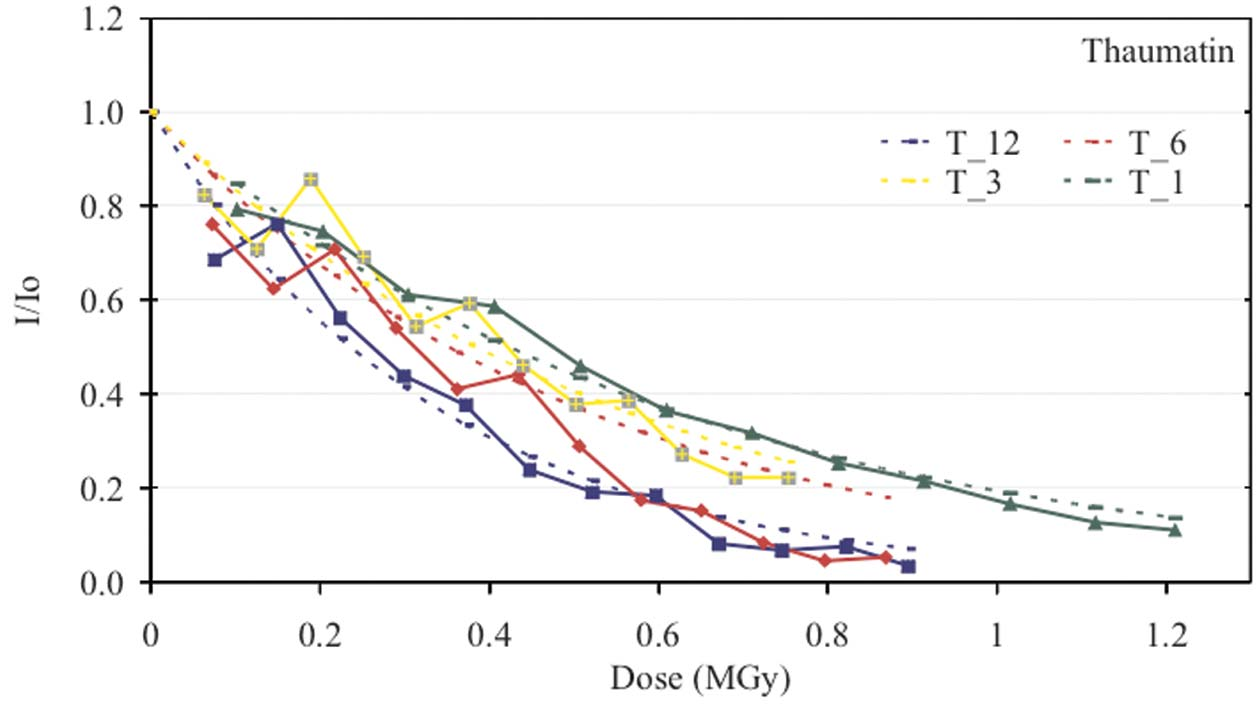
Plot of the relative summed intensity decay as a function of absorbed dose for thaumatin crystals at dose rates T_12 (∼7760 Gy s^−1^, blue), T_6 (∼3770 Gy s^−1^, red), T_3 (∼1635 Gy s^−1^, yellow) and T_1 (∼1320 Gy s^−1^, green). The resolution range is between 4.8 and 1.6 Å.

**Figure 5:**
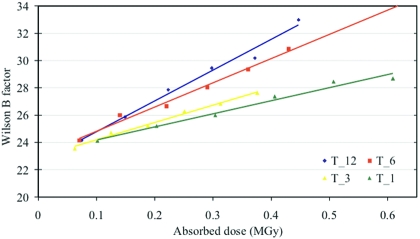
Plot of the change in Wilson *B*-factor of successive datasets for thaumatin crystals T_12, T_6, T_3 and T_1.

**Table 1:** Data acquisition parameters and dose rates

Insulin				Thaumatin			
Dose rate (Gy s^−1^)	Oscillation angle (°)	Flux (10^11^ photons s^−1^)	Crystal	Dose rate (Gy s^−1^)	Oscillation angle (°)	Flux (10^11^ photons s^−1^)	Crystal
∼1430	0.5	0.290	I_1	∼1320	0.5	0.290	T_1
∼1775	0.5	0.359	I_3	∼1635	0.5	0.359	T_3
∼3030	0.5	0.613	I_6	∼3770	0.5	0.613	T_6
∼8420	0.5	1.794	I_12	∼7760	0.5	1.794	T_12

**Table 2:** Data statistics for insulin crystal (measured at 100 K) The resolution range is between 4.14 and 1.39 Å.

Dataset I	Accumulated dose (MGy)	Observed reflections	Multiplicity	〈*I*/σ(*I*)〉	*R*_meas_ (%)	Wilson *B* value (Å^2^)	*I*_mean_	Mosaicity (°)	Δϕ (°)
1	1.4	90658	5.1	33.80	2.7	19.5	875.25	0.086	0.5

**Table 3:** Data statistics for thaumatin crystal (measured at 100 K) The resolution range is between 4.2 and 1.4 Å.

Dataset T	Accumulated dose (MGy)	Observed reflections	Multiplicity	〈*I*/σ(*I*)〉	*R*_meas_ (%)	Wilson *B* value (Å^2^)	*I*_mean_	Mosaicity (°)	Δϕ (°)
1	2.9	325229	6.87	45.06	2.7	17.9	868.87	0.091	0.5

**Table 4:** Data statistics for insulin crystals at elevated temperatures

Dataset	Accumulated dose (MGy)	Observed reflections	Multiplicity	〈*I*/σ(*I*)〉	*R*_meas_ (%)	Wilson *B* value (Å^2^)	*I*_mean_	Mosaicity (°)	*I*/*I*_0_	Resolution range (Å)
I_303	0.09	44922	4.9	46.4	3.0	38.2	157.4	0.074	–	5.0–1.7
I_313	0.09	37642	4.8	41.0	3.5	41.7	218.7	0.087	–	5.3–1.8
I_323	0.09	23071	4.8	42.0	3.0	56.0	146	0.157	–	5.0–2.1
I_333	0.09	10192	5.0	24.1	6.8	92.7	90.0	0.144	–	8.1–2.8
I_343	0.09	9622	4.8	18.9	8.4	95.8	105.5	0.122	1.00	8.3–2.85
I_343-2	0.18	9196	4.6	8.16	18	112	27	0.389	0.26	8.3–2.83

**(a):** At a frame rate of 12.5 Hz.

Dataset I_12	Accumulated dose (MGy)	Observed reflections	Multiplicity	〈*I*/σ(*I*)〉	*R*_meas_ (%)	Wilson *B* value (Å^2^)	*I*_mean_	Mosaicity (°)	*I*/*I*_0_
1	0.06	45414	4.8	18.67	5.8	26.97	198.66	0.028	1.00
2	0.121	40677	4.9	19.81	5.1	30.92	155.75	0.036	0.78
3	0.182	31229	5.0	18.11	5.9	36.16	112.71	0.054	0.57
4	0.242	24688	5.0	18.67	5.9	38.84	73.03	0.076	0.37
5	0.303	20025	5.0	17.36	6.7	40.48	57.30	0.11	0.29
6	0.363	14184	5.0	18.47	7.5	39.63	40.38	0.163	0.20
7	0.424	10500	5.0	13.50	10.7	36.30	23.03	0.23	0.12
8	0.484	7956	4.9	14.64	11.5	34.95	15.78	0.275	0.08

**(b):** At a frame rate of 6.25 Hz.

Dataset I_6	Accumulated dose (MGy)	Observed reflections	Multiplicity	〈*I*/σ(*I*)〉	*R*_meas_ (%)	Wilson *B* value (Å^2^)	*I*_mean_	Mosaicity (°)	*I*/*I*_0_
1	0.044	43982	4.9	21.98	4.6	27.05	99.62	0.02	1.00
2	0.087	42582	4.97	23.56	4.3	28.65	108.56	0.023	1.09
3	0.131	39638	5.0	21.21	4.8	30.65	97.49	0.025	0.98
4	0.174	33313	5.0	18.79	5.6	33.51	61.30	0.031	0.62
5	0.218	28829	5.0	19.02	5.6	36.01	41.63	0.033	0.42
6	0.261	29028	5.1	19.04	5.6	35.98	44.71	0.038	0.45
7	0.305	25160	5.0	18.18	6.2	36.28	39.91	0.053	0.40
8	0.349	17070	5.0	17.23	7.3	38.98	21.23	0.086	0.21

**(c):** At a frame rate of 3.125 Hz.

Dataset I_3	Accumulated dose (MGy)	Observed reflections	Multiplicity	〈*I*/σ(*I*)〉	*R*_meas_ (%)	Wilson *B* value (Å^2^)	*I*_mean_	Mosaicity (°)	*I*/*I*_0_
1	0.051	45279	4.9	26.22	3.9	27.28	139.31	0.023	1.00
2	0.102	42466	5.0	23.51	4.4	29.29	108.92	0.026	0.78
3	0.153	39158	5.0	22.4	4.6	31.37	82.91	0.028	0.60
4	0.204	36809	4.9	22.13	4.7	32.87	75.26	0.029	0.54
5	0.256	32762	5.0	20.51	5.1	34.33	65.25	0.029	0.47
6	0.306	26475	5.1	18.77	5.9	36.43	39.47	0.031	0.28
7	0.357	24298	5.1	19.07	6.1	36.71	28.48	0.036	0.20
8	0.408	25029	5.0	19.3	6	35.95	30.85	0.038	0.22

**(d):** At a frame rate of 1.56 Hz.

Dataset I_1	Accumulated dose (MGy)	Observed reflections	Multiplicity	〈*I*/σ(*I*)〉	*R*_meas_ (%)	Wilson *B* value (Å^2^)	*I*_mean_	Mosaicity (°)	*I*/*I*_0_
1	0.083	46134	5.0	28.47	3.5	27.49	168.97	0.024	1.00
2	0.165	43536	5.0	23.63	4.3	29.94	146.28	0.027	0.87
3	0.247	37612	5.1	20.73	5	32.99	108.85	0.033	0.64
4	0.330	30410	5.0	23.30	4.5	36.34	82.59	0.045	0.49
5	0.413	25696	5.0	26.04	4.4	38.83	63.19	0.065	0.37
6	0.495	18228	5.1	13.91	9.1	41.45	49.45	0.109	0.29
7	0.578	12071	5.1	12.13	10.9	39.37	33.71	0.175	0.20
8	0.660	9051	5.1	13.83	10	36.29	19.99	0.224	0.12

**(a):** At a frame rate of 12.5 Hz.

Dataset T_12	Accumulated dose (MGy)	Observed reflections	Multiplicity	〈*I*/σ(*I*)〉	*R*_meas_ (%)	Wilson *B* value (Å^2^)	*I*_mean_	Mosaicity (°)	*I*/*I*_0_
1	0.075	125855	4.2	22.35	4.3	24.16	217.27	0.037	1.00
2	0.149	125103	4.3	20.45	4.9	25.86	241.71	0.041	1.11
3	0.223	115807	4.3	18.47	5.4	27.86	177.92	0.045	0.82
4	0.298	108840	4.4	18.28	5.6	29.45	139.41	0.062	0.64
5	0.372	100527	4.4	18.74	5.6	30.18	120.55	0.076	0.56
6	0.447	72258	4.3	18.49	6.4	32.99	75.07	0.109	0.35
7	0.521	69073	4.6	19.67	6.2	32.91	60.08	0.134	0.28
8	0.596	63866	4.4	16.72	7.4	33.14	59.34	0.145	0.27
9	0.670	35686	4.3	13.35	11.4	33.72	26.10	0.209	0.12
10	0.745	39141	4.7	14.59	10.7	33.07	21.88	0.199	0.10
11	0.821	43755	4.5	14.82	10	32.84	23.12	0.194	0.11
12	0.894	25791	3.7	9.47	16	32.19	10.33	0.183	0.05

**(b):** At a frame rate of 6.25 Hz.

Dataset T_6	Accumulated dose (MGy)	Observed reflections	Multiplicity	〈*I*/σ(*I*)〉	*R*_meas_ (%)	Wilson *B* value (Å^2^)	*I*_mean_	Mosaicity (°)	*I*/*I*_0_
1	0.07	122491	4.3	21.42	4.7	24.16	105.18	0.024	1.00
2	0.14	118403	4.3	21.41	4.9	25	86.06	0.025	0.82
3	0.22	114839	4.37	21.03	4.9	26.67	97.97	0.026	0.93
4	0.29	108029	4.38	19.96	5.1	28.05	75.04	0.027	0.71
5	0.36	98274	4.35	19.41	5.5	29.38	56.77	0.028	0.54
6	0.43	91389	4.4	18.39	5.7	30.87	61.10	0.034	0.58
7	0.51	69756	4.5	18.21	6.5	33.41	39.68	0.074	0.38
8	0.58	42819	4.3	18.14	8	34.32	24.30	0.153	0.23
9	0.65	33605	4.4	16.53	9.5	33.59	21.05	0.231	0.20
10	0.72	24955	4.5	12.51	12.7	31.35	11.07	0.217	0.11
11	0.8	43000	4	5.04	26.3	20.66	6.46	0.197	0.06
12	0.87	24797	4.6	10.05	15.9	27.45	7.22	0.202	0.07

**(c):** At a frame rate of 3.125 Hz.

Dataset T_3	Accumulated dose (MGy)	Observed reflections	Multiplicity	〈*I*/σ(*I*)〉	*R*_meas_ (%)	Wilson *B* value (Å^2^)	*I*_mean_	Mosaicity (°)	*I*/*I*_0_
1	0.063	130817	4.3	25.75	3.8	23.54	199.39	0.027	1.00
2	0.125	130517	4.4	24	4.2	24.71	172.13	0.027	0.86
3	0.188	129075	4.3	21.09	4.7	25.22	207.76	0.032	1.042
4	0.251	124326	4.4	24.25	4	26.27	167.57	0.032	0.84
5	0.313	121683	4.47	24.52	4.1	26.82	132.21	0.031	0.66
6	0.376	118557	4.3	23.48	4.2	27.63	143.25	0.031	0.72
7	0.439	111192	4.49	19.2	5.1	28.64	112.00	0.036	0.56
8	0.502	108024	4.53	24	4.4	29.06	91.78	0.06	0.46
9	0.564	92996	4.36	21.4	5.4	29.83	93.31	0.091	0.47
10	0.627	79184	4.67	16.83	6.8	30.71	65.08	0.117	0.33
11	0.690	76800	4.5	19.88	6.5	30.28	53.53	0.128	0.27
12	0.753	63514	4.35	19.42	6.9	31.50	53.04	0.151	0.27

**(d):** At a frame rate of 1.56 Hz.

Dataset T_1	Accumulated dose (MGy)	Observed reflections	Multiplicity	〈*I*/σ(*I*)〉	*R*_meas_ (%)	Wilson *B* value (Å^2^)	*I*_mean_	Mosaicity (°)	*I*/*I*_0_
1	0.102	133906	4.29	31.54	3.1	24.12	292.07	0.034	1.00
2	0.203	131302	4.37	30.29	3.2	25.19	273.26	0.037	0.94
3	0.304	127007	4.3	28.01	3.5	25.99	224.57	0.059	0.77
4	0.406	122687	4.33	25.15	3.8	27.36	214.86	0.062	0.74
5	0.507	116650	4.37	27.29	3.6	28.46	168.22	0.056	0.58
6	0.609	111102	4.34	26.78	3.8	28.68	134.43	0.062	0.46
7	0.711	106271	4.39	26.59	3.8	29.69	116.04	0.057	0.39
8	0.812	97757	4.41	24.6	4.3	30.44	94.21	0.07	0.32
9	0.914	81509	4.37	22.54	5.4	31.85	78.82	0.101	0.27
10	1.015	65428	4.43	21.21	5.7	33.52	61.01	0.125	0.21
11	1.116	57091	4.37	21.04	6.1	34.79	47.57	0.137	0.16
12	1.21	49840	4.32	19.22	7.3	33.62	40.88	0.163	0.14

**Table 7:** Linear fit analysis of the *B*-factor *versus* dose rates *B*_1_ denotes the values of the first data set in a series.

Insulin				Thaumatin			
Dose rate (Gy s^−1^)	Δ*B*/Δ*D* (Å^2^ MGy^−1^)	*B*_1_ (Å^2^)	R-Sq	Dose rate (Gy s^−1^)	Δ*B*/Δ*D* (Å^2^ MGy^−1^)	*B*_1_ (Å^2^)	R-Sq
∼1430	35.2	24.3	0.99	∼1320	9.09	23.3	0.98
∼1775	34.6	25.7	0.99	∼1635	12.3	22.9	0.99
∼3030	52.2	24.3	0.98	∼3770	18.1	23	0.95
∼8420	57.5	24.2	0.95	∼7760	22.5	23.8	0.92

## Footnotes

1 Supplementary data for this paper are available from the IUCr electronic archives (Reference: XH5025). Services for accessing these data are described at the back of the journal.

2 The true mosaicity may be considerably lower, because the oscillation angle of 0.5° results in an insufficient number of partial reflections to estimate the mosaicity.

3 The intrinsic mosaicity of protein crystals at RT can be significantly narrower than refined by integration programs. Reciprocal-space mapping of lysozyme crystals revealed an instrument-limited reflection width of 0.0015° (Schulze-Briese *et al.*, 1999 ▶).

## References

[bb1] Adams, P. D., Afonine, P. V., Bunkóczi, G., Chen, V. B., Davis, I. W., Echols, N., Headd, J. J., Hung, L.-W., Kapral, G. J., Grosse-Kunstleve, R. W., McCoy, A. J., Moriarty, N. W., Oeffner, R., Read, R. J., Richardson, D. C., Richardson, J. S., Terwilliger, T. C. & Zwart, P. H. (2010). *Acta Cryst.* D**66**, 213–221. 10.1107/S0907444909052925 PMC2815670 20124702

[bb2] Alcorn, T. & Juers, D. H. (2010). *Acta Cryst.* D**66**, 366–373. 10.1107/S090744490903995X PMC2852300 20382989

[bb3] Barker, A. I., Southworth-Davies, R. J., Paithankar, K. S., Carmichael, I. & Garman, E. F. (2009). *J. Synchrotron Rad.* **16**, 205–216. 10.1107/S0909049509003343 19240332

[bb4] Berthold, C. L., Toyota, C. G., Richards, N. G. & Lindqvist, Y. (2008). *J. Biol. Chem.* **283**, 6519–6529. 10.1074/jbc.M709353200 18162462

[bb5] Bingel-Erlenmeyer, R., Olieric, V., Grimshaw, J. P. A., Gabadinho, J., Wang, X., Ebner, S. G., Isenegger, A., Schneider, R., Schneider, J., Glettig, W., Pradervand, C., Panepucci, E. H., Tomizaki, T., Wang, M. & Schulze-Briese, C. (2011). *Cryst. Growth Des.* Accepted.

[bb6] Blake, C. & Phillips, D. C. (1962). *Proceedings of the Symposium on the Biological Effects of Ionizing Radiation at the Molecular Level (Vienna:International Atomic Energy Agency)*, pp. 183–191.

[bb7] Bourgeois, D. & Royant, A. (2005). *Curr. Opin. Struct. Biol.* **15**, 538–547. 10.1016/j.sbi.2005.08.002 16129597

[bb8] Cherezov, V., Riedl, K. M. & Caffrey, M. (2002). *J. Synchrotron Rad.* **9**, 333–341. 10.1107/s0909049502014528 12409619

[bb9] Colletier, J. P., Bourgeois, D., Sanson, B., Fournier, D., Sussman, J. L., Silman, I. & Weik, M. (2008). *Proc. Natl Acad. Sci. USA*, **105**, 11742–11747. 10.1073/pnas.0804828105 PMC2575335 18701720

[bb10] Dinapoli, R. (2011). *PIXEL2010*, Grindelwald, Switzerland, *Nucl. Instrum. Methods Phys. Res. A.* Submitted.

[bb11] Dodson, E. J., Dodson, G. G., Lewitova, A. & Sabesan, M. (1978). *J. Mol. Biol.* **125**, 387–396. 10.1016/0022-2836(78)90409-6 731699

[bb12] Evans, P. (2006). *Acta Cryst.* D**62**, 72–82. 10.1107/S0907444905036693 16369096

[bb13] Garman, E. (1999). *Acta Cryst.* D**55**, 1641–1653. 10.1107/s0907444999008653 10531512

[bb14] Henderson, R. (1990). *Proc. R. Soc. London Ser. B*, **241**, 6–8.

[bb15] Hendrickson, W. A. (1976). *J. Mol. Biol.* **106**, 889–893. 10.1016/0022-2836(76)90271-0 978739

[bb16] Hülsen, G., Broennimann, C., Eikenberry, E. F. & Wagner, A. (2006). *J. Appl. Cryst.* **39**, 550–557.

[bb17] Huus, K., Havelund, S., Olsen, H. B., van de Weert, M. & Frokjaer, S. (2005). *Biochemistry*, **44**, 11171–11177. 10.1021/bi0507940 16101301

[bb18] Juers, D. H. & Matthews, B. W. (2004). *Q. Rev. Biophys.* **37**, 105–119. 10.1017/s0033583504004007 15999418

[bb19] Kabsch, W. (2010). *Acta Cryst.* D**66**, 125–132. 10.1107/S0907444909047337 PMC2815665 20124692

[bb20] Kalinin, Y., Kmetko, J., Bartnik, A., Stewart, A., Gillilan, R., Lobkovsky, E. & Thorne, R. (2005). *J. Appl. Cryst.* **38**, 333–339.

[bb21] Kmetko, J., Husseini, N. S., Naides, M., Kalinin, Y. & Thorne, R. E. (2006). *Acta Cryst.* D**62**, 1030–1038. 10.1107/S0907444906023869 16929104

[bb22] Ko, T.-P., Day, J., Greenwood, A. & McPherson, A. (1994). *Acta Cryst.* D**50**, 813–825. 10.1107/S0907444994005512 15299348

[bb23] Kriminski, S., Caylor, C. L., Nonato, M. C., Finkelstein, K. D. & Thorne, R. E. (2002). *Acta Cryst.* D**58**, 459–471. 10.1107/s0907444902000112 11856832

[bb24] Kurinov, I. V. & Harrison, R. W. (1995). *Acta Cryst.* D**51**, 98–109. 10.1107/S0907444994009261 15299341

[bb25] Leiros, H.-K. S., Timmins, J., Ravelli, R. B. G. & McSweeney, S. M. (2006). *Acta Cryst.* D**62**, 125–132. 10.1107/S0907444905033627 16421442

[bb26] Lovelace, J. J., Murphy, C. R., Pahl, R., Brister, K. & Borgstahl, G. E. O. (2006). *J. Appl. Cryst.* **39**, 425–432.

[bb27] McFerrin, M. B. & Snell, E. H. (2002). *J. Appl. Cryst.* **35**, 538–545.

[bb28] McGeehan, J., Ravelli, R. B. G., Murray, J. W., Owen, R. L., Cipriani, F., McSweeney, S., Weik, M. & Garman, E. F. (2009). *J. Synchrotron Rad.* **16**, 163–172. 10.1107/S0909049509001629 PMC2651762 19240328

[bb29] Meents, A., Gutmann, S., Wagner, A. & Schulze-Briese, C. (2010). *Proc. Natl Acad. Sci. USA*, **107**, 1094–1099. 10.1073/pnas.0905481107 PMC2798883 20080548

[bb30] Müller, M. (2010). Personal communication.

[bb32] Murray, J. W., Garman, E. F. & Ravelli, R. B. G. (2004). *J. Appl. Cryst.* **37**, 513–522.

[bb33] Nave, C. & Garman, E. F. (2005). *J. Synchrotron Rad.* **12**, 257–260. 10.1107/S0909049505007132 15840908

[bb34] Sauter, N. K., Grosse-Kunstleve, R. W. & Adams, P. D. (2004). *J. Appl. Cryst.* **37**, 399–409. 10.1107/S0021889804005874 PMC2808709 20090869

[bb35] Owen, R. L., Holton, J. M., Schulze-Briese, C. & Garman, E. F. (2009*a*). *J. Synchrotron Rad.* **16**, 143–151. 10.1107/S0909049508040429 PMC2651761 19240326

[bb36] Owen, R. L., Pearson, A. R., Meents, A., Boehler, P., Thominet, V. & Schulze-Briese, C. (2009*b*). *J. Synchrotron Rad.* **16**, 173–182. 10.1107/S0909049508040120 PMC2651763 19240329

[bb37] Owen, R. L., Rudiño-Piñera, E. & Garman, E. F. (2006). *Proc. Natl Acad. Sci. USA*, **103**, 4912–4917. 10.1073/pnas.0600973103 PMC1458769 16549763

[bb38] Paithankar, K. S., Owen, R. L. & Garman, E. F. (2009). *J. Synchrotron Rad.* **16**, 152–162. 10.1107/S0909049508040430 19240327

[bb39] Ravelli, R. B. G., Theveneau, P., McSweeney, S. & Caffrey, M. (2002). *J. Synchrotron Rad.* **9**, 355–360. 10.1107/s0909049502014541 12409622

[bb40] Rey, F. (2011). Personal communication.

[bb41] Sanchez-Weatherby, J., Bowler, M. W., Huet, J., Gobbo, A., Felisaz, F., Lavault, B., Moya, R., Kadlec, J., Ravelli, R. B. G. & Cipriani, F. (2009). *Acta Cryst.* D**65**, 1237–1246. 10.1107/S0907444909037822 19966409

[bb42] Schneider, G. (2008). Personal communication.

[bb44] Schulze-Briese, C., Pradervand, C., Janousch, M., Briand, C., Tschopp, M., Grütter, M., Storici, P. & Schirmer, T. (1999). *PSI Annu. Rep.* **VII**, 31.

[bb43] Schulze-Briese, C., Wagner, A., Tomizaki, T. & Oetiker, M. (2005). *J. Synchrotron Rad.* **12**, 261–267. 10.1107/S0909049505003298 15840909

[bb45] Sliz, P., Harrison, S. C. & Rosenbaum, G. (2003). *Structure*, **11**, 13–19. 10.1016/s0969-2126(02)00910-3 12517336

[bb46] Snell, E. H., Bellamy, H. D., Rosenbaum, G. & van der Woerd, M. J. (2007). *J. Synchrotron Rad.* **14**, 109–115. 10.1107/S090904950604605X 17211077

[bb47] Southworth-Davies, R. J., Medina, M. A., Carmichael, I. & Garman, E. F. (2007). *Structure*, **15**, 1531–1541. 10.1016/j.str.2007.10.013 18073104

[bb48] Stuart, D. (2011). Personal communication.

[bb49] Teng, T. & Moffat, K. (2000). *J. Synchrotron Rad.* **7**, 313–317. 10.1107/S0909049500008694 16609214

[bb50] Teng, T.-Y. & Moffat, K. (2002). *J. Synchrotron Rad.* **9**, 198–201. 10.1107/s0909049502008579 12091725

[bb51] Wang, M. (2011). Personal communication.

[bb52] Warkentin, M. & Thorne, R. E. (2010). *Acta Cryst.* D**66**, 1092–1100. 10.1107/S0907444910035523 PMC2954455 20944242

