# Supplementary material for: Radiation damage in room-temperature data acquisition with the PILATUS 6M pixel detector
Source: J Synchrotron Radiat. 2011 Apr 9;18(Pt 3):318–28. doi: 10.1107/S090904951100968X (PMC3133521; doi:10.1107/S090904951100968X)
Supplement: Supplementary file 1 [file s-18-00318-sup1.pdf]

Supplementary material - 1

Sample heating experiment

Data were collected at elevated temperatures ranging from 25°C to 80°C. A conventional hot gun was used to blow warm air at the sample. The temperature of the sample was measured with a thermocouple which was kept within ~3 mm distance from the sample. The state of the insulin crystals before and after data collection at the different temperatures as observed with the sample alignment microscope is shown in figure S1. Each data set corresponds to 45° (90 images of 0.5° oscillation angle), with 0.64 s / frame exposure time at a flux of ~2.5 to 2.9 × 10^{10} ph / sec. The beam size was 100 µm by 80 µm. These parameters correspond to a dose of ~8.9 × 10^{4} Gy per data set

Figure S1 Pictures showing the state of insulin crystals before (pre) and after (post) high temperature data collection

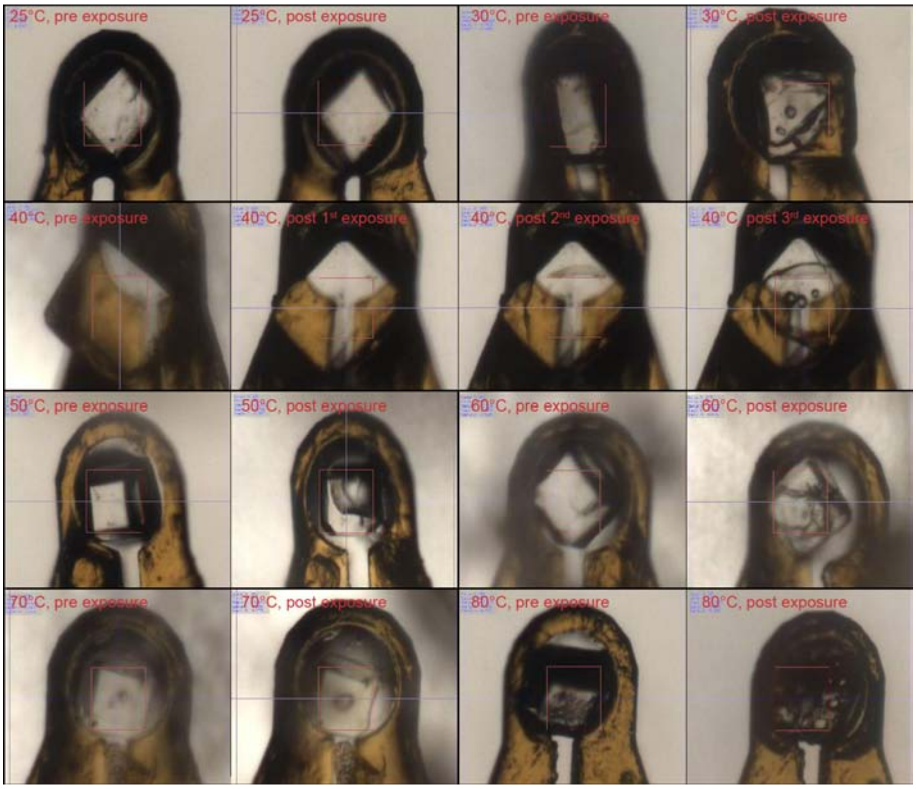

Supplementary material-2

Figure S2

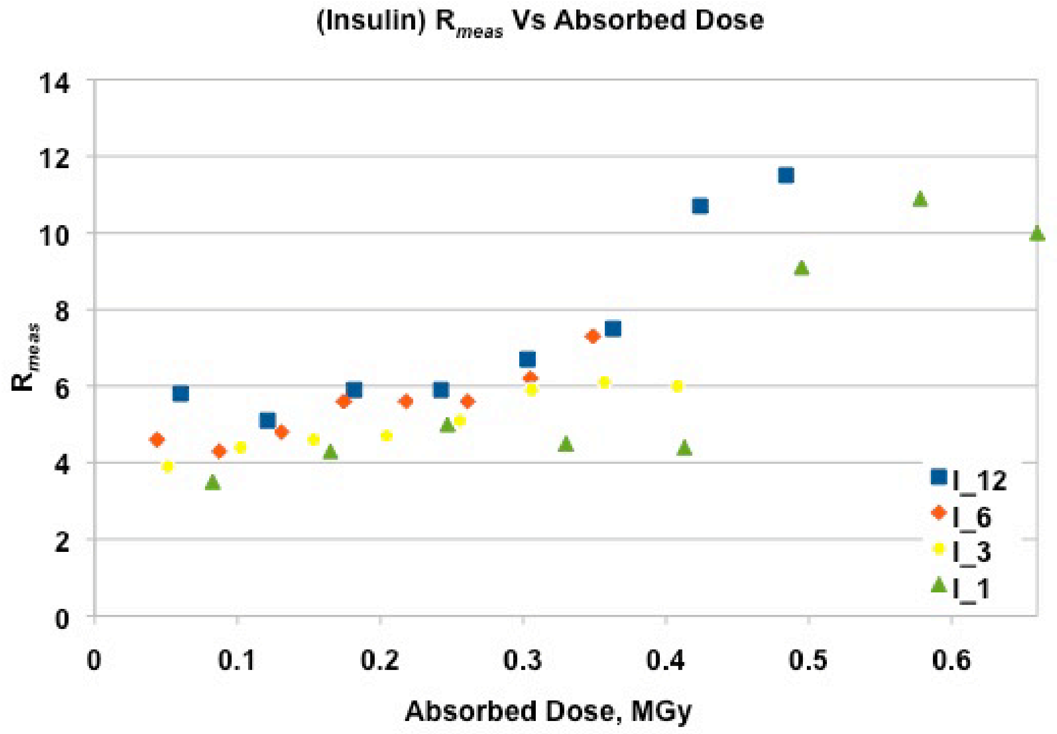

Figure S3

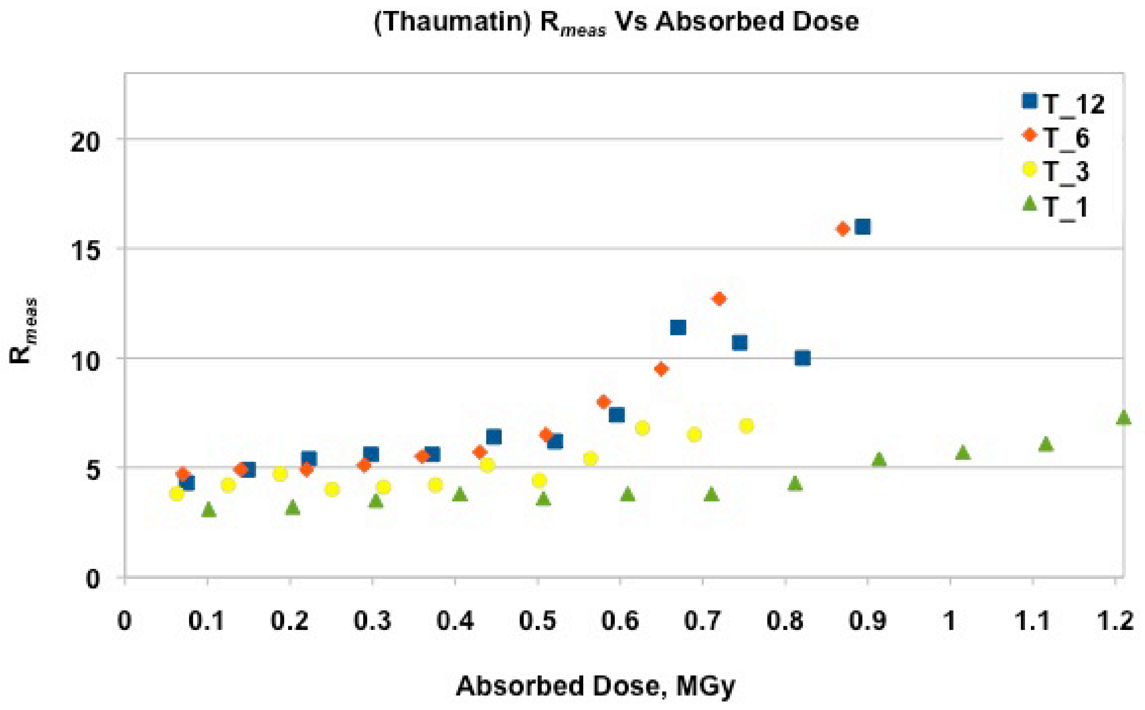

Figure S4

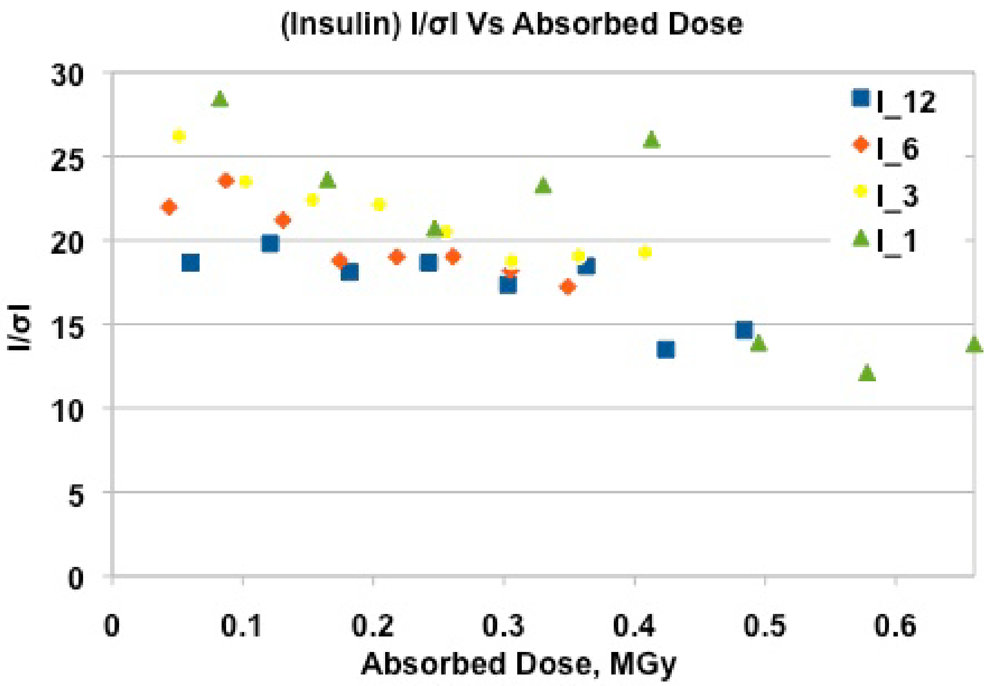

Figure S5

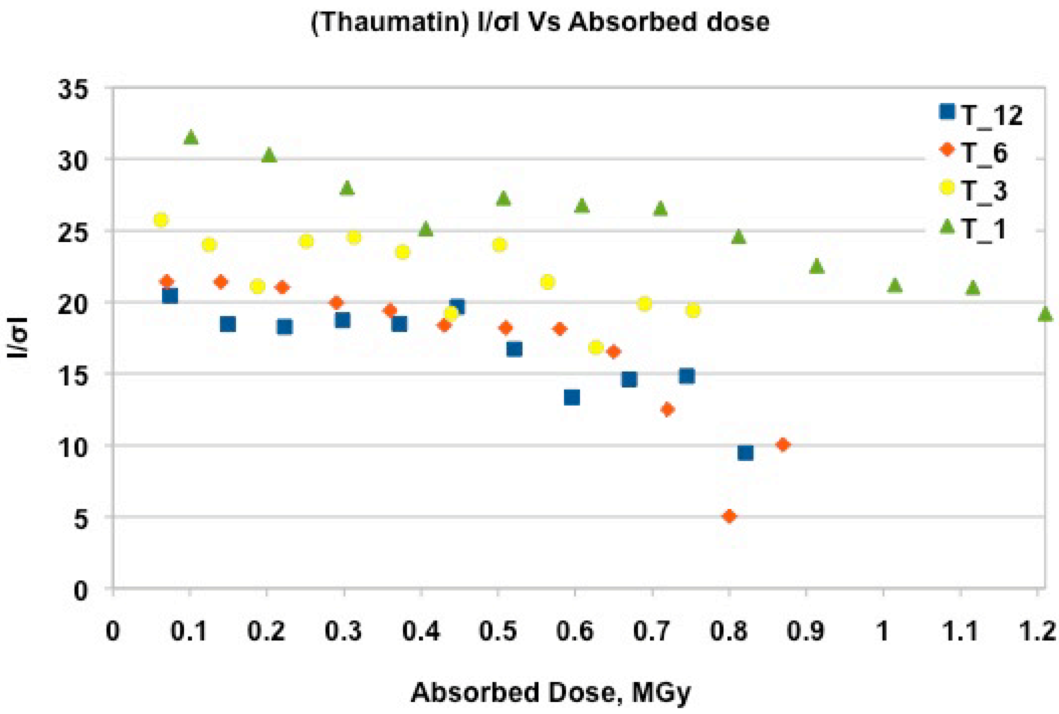

Figure S6

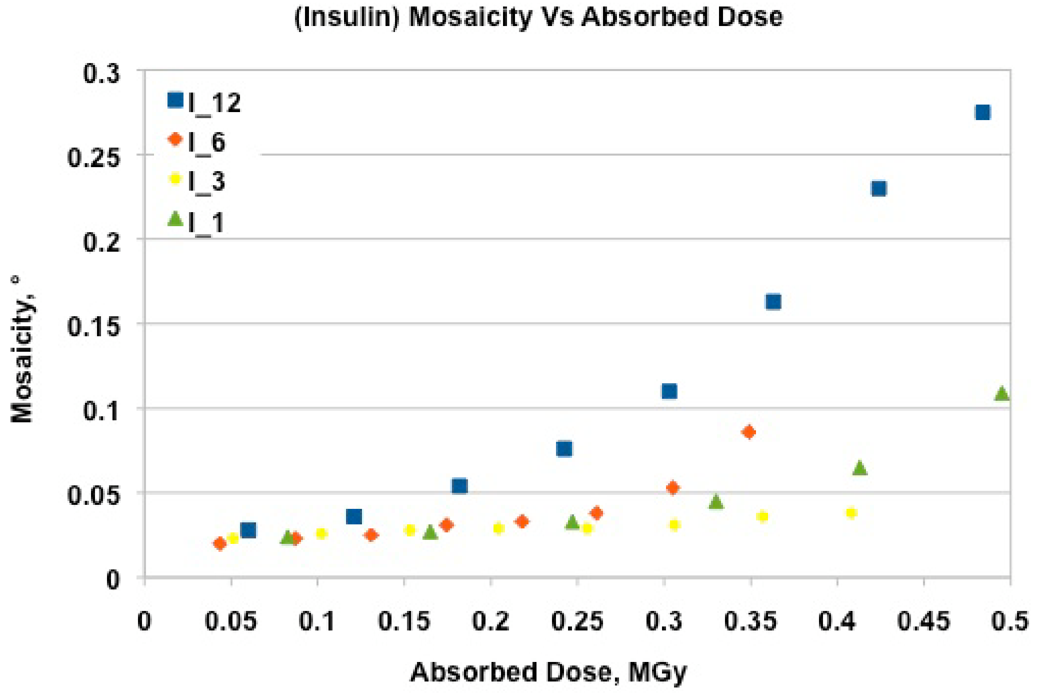
(Insulin) Mosaicity Vs Absorbed Dose
I_12
I_6
I_3
I_1
Mosaicity, °
0.3
0.25
0.2
0.15
0.1
0.05
0
0 0.05 0.1 0.15 0.2 0.25 0.3 0.35 0.4 0.45 0.5
Absorbed Dose, MGy

Figure S7

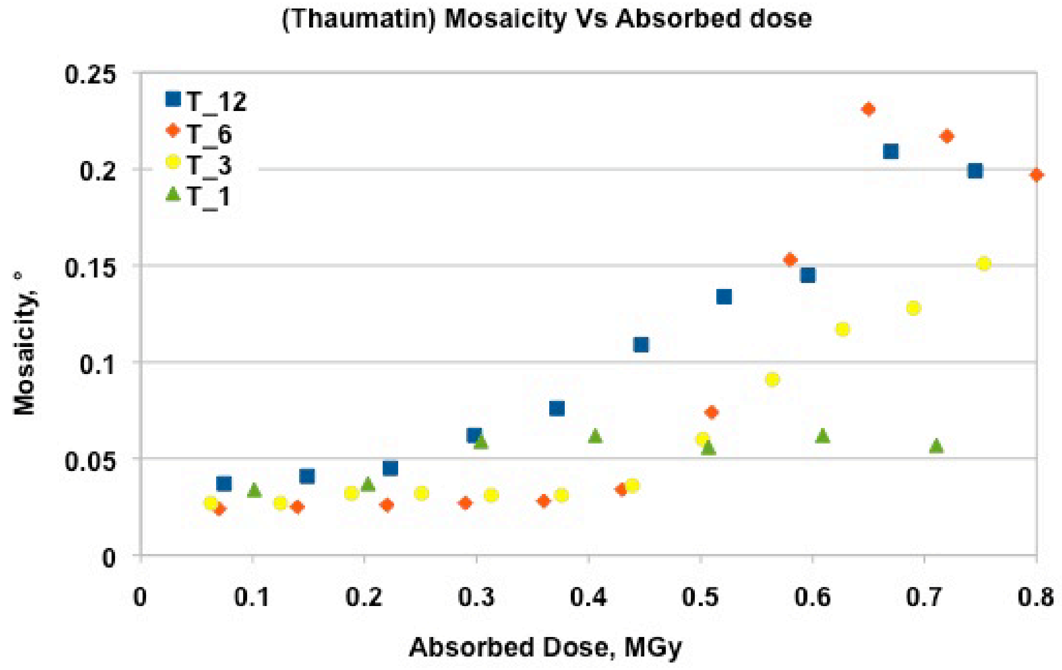
(Thaumatin) Mosaicity Vs Absorbed dose
T_12
T_6
T_3
T_1
Mosaicity, °
0.25
0.2
0.15
0.1
0.05
0
0 0.1 0.2 0.3 0.4 0.5 0.6 0.7 0.8
Absorbed Dose, MGy

Figure S2
Plot of the change in R_{meas} of successive datasets for insulin crystals I_12, I_6, I_3 and I_1. The resolution range used for the calculation is between 4.8 – 1.6 Å.

Figure S3
Plot of the change in R_{meas} of successive datasets for thaumatin crystals T_12, T_6, T_3 and T_1. The resolution range used for the calculation is between 4.8-1.6 Å

Figure S4
Plot of the change in I/σI of successive datasets for insulin crystals I_12, I_6, I_3 and I_1. The resolution range used for the calculation is between 4.8 – 1.6 Å.

Figure S5
Plot of the change in I/σI of successive datasets for thaumatin crystals T_12, T_6, T_3 and T_1. The resolution range used for the calculation is between 4.8-1.6 Å.

Figure S6
Plot of the change in mosaicity of successive datasets for insulin crystals I_12, I_6, I_3 and I_1. The resolution range used for the calculation is between 4.8 – 1.6 Å.

Figure S7
Plot of the change in mosaicity of successive datasets for thaumatin crystals T_12, T_6, T_3 and T_1. The resolution range used for the calculation is between 4.8-1.6 Å

Supplementary material -3

UV-Vis absorption spectroscopy experiments

The change in optical density (OD) in the visible range of an insulin crystal irradiated by ten X-ray beam pulses (where each is of 1 s length and separated by 4 s without irradiation) was measured by means of the co-axial microspectrophotometer installed at SLS beamline X10SA (Owen *et al.*, 2009). The X-ray beam size was ~110 µm by 90 µm, while the spot size of the microspectrophotometer illumination was 50 µm by 50 µm. Absorption data were recorded using an ANDOR Shamrock Czerny-Turner spectrograph, using entrance slit sizes of 10 µm and an optical grating with 150 lines/mm, blazed at 300 nm. Kinetic spectra were acquired with an acquisition time of 3.42 ms and cycle frequency of 48.92 Hz (20.44 ms). The dose per second of the X-ray beam irradiation was calculated using *RADDOSE* (Murray *et al.*, 2004) to be 9×10^3 Gy. The temporal evolution of the signal at 400 nm and 600 nm was measured at 100 K (Figure S8a) and at 298 K (Figure S8c). The absorbance at 400 nm and 600 nm corresponds to disulfide radical anions and trapped electrons respectively (McGeehan *et al.*, 2009). At 100 K we could not observe any signature of trapped electrons. No indication of either of the radiolytic products could be detected at RT (Figure S8c).

Figure S8

The optical density of an insulin crystal irradiated for 10 beam pulses of 1s each, separated by 4 s without X-ray irradiation, followed at 400 nm and from 520-620 nm in 20 nm steps at 100 K (Figure S8 a & b) and RT (Figure S8 c)

Figure S8

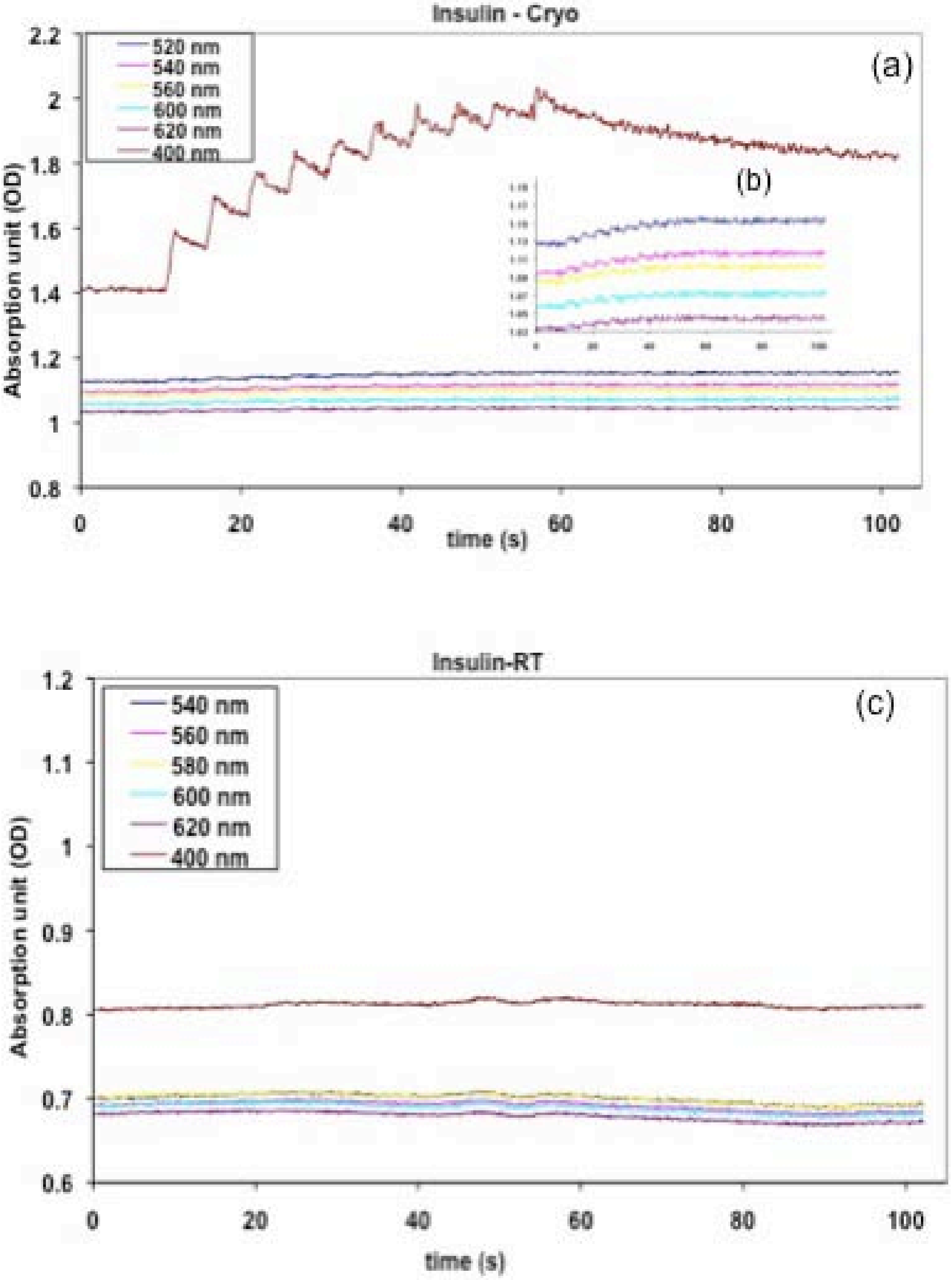

Insulin - Cryo
(a)
(b)
520 nm
540 nm
560 nm
600 nm
620 nm
400 nm
Absorption unit (OD)
2.2
2
1.8
1.6
1.4
1.2
1
0.8
0
20
40
60
80
100
time (s)
Insulin-RT
(c)
540 nm
560 nm
580 nm
600 nm
620 nm
400 nm
Absorption unit (OD)
1.2
1.1
1
0.9
0.8
0.7
0.6
0
20
40
60
80
100
time (s)

Supplementary material-4

Figure S9

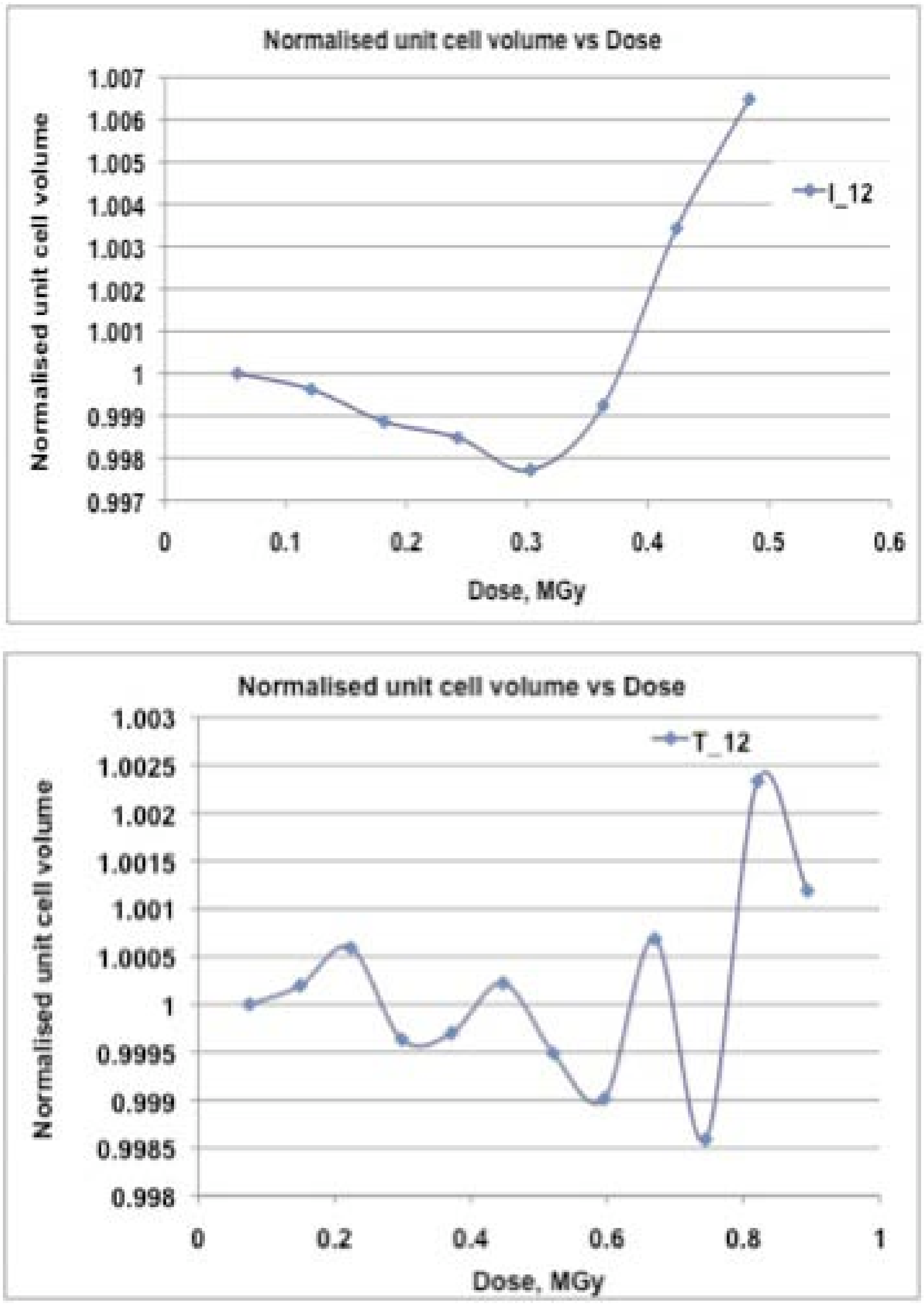

Figure S9: Plot showing normalized unit cell volume vs. dose at a frame rate of 12.5 Hz for insulin (I_12) and thaumatin (T_12) crystal respectively

Figure S10

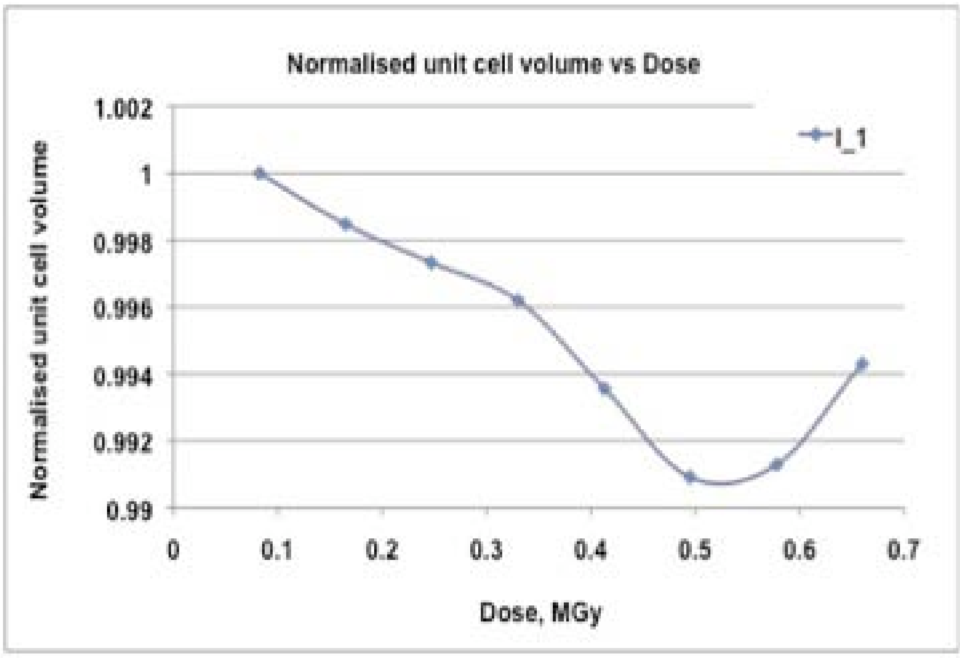

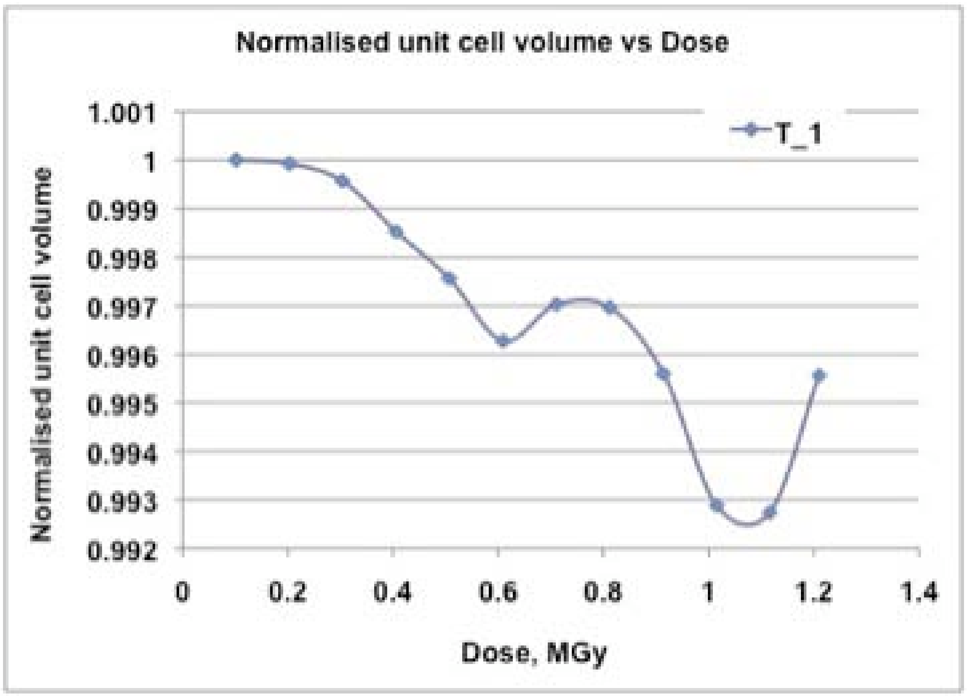

Figure S10: Plot showing normalized unit cell volume vs. dose at a frame rate of 1.5625 Hz for insulin (I_1) and thaumatin (T_1) crystal respectively
